# Thirteenth International Medical Congress, Paris, August, 1900. (*Abstracts*.)

**Published:** 1900-10

**Authors:** A. H. Ward

**Affiliations:** F. R. C. S., England. Surgeon to the London Lock Hospital


### Society Proceedings.

### THIRTEENTH INTERNATIONAL MEDICAL CONGRESS, PARIS, AUGUST, 1900.

(*Abstracts.*)

### CAUSES OF THE GENERALISED INFECTIONS BY THE GONOCOCCUS.

By A. H. WARD, F. R. C. S., England.
Surgeon to the London Lock Hospital.

THE generalisation of the gonorrheal infection cannot be logically separated from the local focus whence it originates. There is a continuous interaction, and the persistence of the local disease aggravates the general condition. The whole process of the struggle for life between the microbe, and the human organism, has to be studied, and from the facts thus collected, a plan of action for prevention and cure, deduced. Whether the diplococcus of Neisser and its toxin, is the sole cause of the local and general disease, or whether this infection merely paves the way for other organisms, is still an open question. The fact remains, that gonorrhea and its associated pathological states, are due to the presence of microbes and their toxic products. The particular microbes, their names and staining reactions are questions for the final decision of the bacteriologist and laboratory expert. The reporter has no personal excursions into these special realms to bring forward, but will endeavor to synthesise the observations of many specialists, in the light of clinical experience, and so build up a coherent conception of the life history of the infection in relation to man.

*The struggle for life between the microbes and the body.*—The thesis advanced is as follows: the gonococcus in its process of growth in the body produces an irritating toxin, which is the direct cause of all symptoms, both local and general, in every case the toxin is absorbed into the system, where it causes systemic degenerations of varying degrees of severity. Gonorrhea is thus a general toxemic affection, but the microbes which form the toxin are generally localised in, or around, a mucous tract. The infection may spread by continuity to the ducts and organs which communicate with the tract affected, or it may penetrate to the interior of the body, either by direct extension, or by a process of growth through the mucous membrane affected. Thence the invasion reaches the cellular tissues, the lymphatics and glands, and the vascular system. This invasion is rendered possible by the paralysing effect of the toxin absorbed upon the leucocytes, which in susceptible individuals, hinders the process of phagocytosis. Having reached the circulation, the microbes are conveyed to the heart, or to terminal capillary circulations in serous and synovial membranes, or to the slender vessels in tendons and fibrous structures. In these situations they become stranded, and develop, forming more toxin, which sets up local inflammations. The invasion of the organism is favored by all too-energetic measures, directed to the local infection, since these depress the local powers of resistance; and by abraiding or lacerating the mucous surface, may directly open doors to the invaders. General treatment should be directed to the general toxemia when present alone, or to the toxemia complicated by metastases. Local treatment is always required, and should be free from instrumental, mechanical, or chemical violence.

*The toxin of the gonococcus and its absorption.*—The demonstration of its existence and properties. Its absorption the cause of the malaise, temperature and anemia, seen in all severe cases. The local discharge is the osmotic and chemotaxis, equivalent of the toxin absorbed. Grave general toxemic degenerations occur in exceptional cases. Cachexia, nervous affections, optic neuritis, erythematous, hemorrhagic, and hyperkeratitic skin diseases are the signs of such degenerative processes. The distinction between these general toxic effects and the local inflammations caused by metastases.

*The invasion of the tissues by the gonococcus.*—This invasion only possible, when the leucocytes are inhibited by the toxin absorbed from the primary focus. The action of local antiseptic measures.

*The action of the toxin on the leucocytes.*—Drobny's researches, Heiman's case. Brewer's views on the invasion of the lymphatic system.

*The gonococcus in the blood.*—Demonstration by Panichi, by the cultivation method. The invasion of the joints generally sparse, and followed by chronic inflammations. Cardiac disease.

*The local effects of the toxin developed in metastases.*—Reinforced by the toxin absorbed from the primary focus. The destruction of this focus necessary in all cases.

*Prevention and cure.*—General toxemia attacked by measures directed to the elimination, or attenuation of the toxin. Metastases attacked by germicides. The dangers of local violence. A case in point. The reporter's experience of generalisations—12 in 2,000 cases or 0.6 per 100. Neisser's words re-echoed: "It is surely beyond a doubt that the frequency of complications depends on the method of treatment."

---

### TREATMENT OF CARCINOMA OF THE UTERUS.

By THOMAS S. CULLEN, M. D., Baltimore.

The speaker briefly referred to the gradual improvement in the operation for carcinoma of the uterus, commencing with amputation of the cervix as practised by Schröder and his contemporaries, abdominal hysterectomy as recommended by Freund, the catheterisation of the ureters as employed by Pawlick and Kelly and the more radical operation consisting in the removal of the iliac glands as advocated by Ries, Rumpf and Clark. Cullen then pointed out the danger of implanting carcinomatous into healthy tissue as is very likely to occur when the radical operation is performed. He then described in detail the operation as performed by Werder, of Pittsburg. In this operation the chance of implanting carcinomatous tissue is reduced to a minimum. The various stages in the abdominal operation, where the cancer is limited to the cervix, are as follows:

1. Removal of broken down carcinomatous cervical tissue, preferably a few days before.
2. Insertion of ureteral bougies if desired.
3. Ligation of the ovarian vessels and round ligaments.
4. Freeing of the bladder from the uterus and broad ligaments.
5. Opening of broad ligaments, location and freeing of the ureters to the points at which they enter the bladder.
6. Ligation of the uterine vessels near their points of origin.
7. Dissection of bladder free from vagina vault.
8. — rectum from — —
9. Removal of pelvic lymph glands.
10. Freeing of vaginal fornices.
11. Closure of pelvic cavity by uniting the vesical peritoneum with that of the rectum, an assistant meanwhile making strong traction on the cervix from below.
12. Closure of the abdomen.

13. Ringing of the vaginal vault with a thermo-cautery or knife, thus freeing the uterus and its surrounding vaginal mucosa.

14. Application of a light gauze pack to the space left in the vaginal vault.

As will be noted the uterus is freed on all sides, the vagina dissected loose from the bladder and rectum and the pelvic and abdominal cavities closed before the operator comes in contact with the carcinomatous cervix. Even then there is little or no danger of transplantation of carcinomatous tissue. Cullen advised this operation in all cases where the patient is not excessively stout. Of course in such instances we have frequently to rely on a vaginal operation entirely. In adeno-carcinoma of the body of the uterus the same operation may be performed, but the wide removal of the vaginal mucosa is not necessary. The speaker then briefly analysed 176 cases of carcinoma of the uterus occurring in the Johns Hopkins Hospital during the last six years.

General summary of the cases of carcinoma of the uterus:

| Variety of Carcinoma. | Operative cases. | Patients well January 1, 1900. |
|---|---|---|
| Squamous cell carcinoma of the cervix | 61 | 13–21 per cent. |
| Adeno-carcinoma of the cervix | 12 | 2–16 per cent. |
| Adeno-carcinoma of the body of the uterus | 30 | 19–63 per cent. |

Cases coming too late for operation.

| | |
|---|---|
| Squamous cell carcinoma of the cervix | 62 cases. |
| Adeno-carcinoma of the cervix | 6 cases. |
| Adeno-carcinoma of the body | 5 cases. |
| Total | 76 cases. |

The prognosis is most favorable in adeno-carcinoma of the body; least promising in adeno-carcinoma of the cervix.

---

### ARTIFICIAL FOODS.

By Prof. A. JACOBI, M. D., L.L. D., New York.

The results of the analyses of human milk are contradictory, no two are alike. Alterations in different periods of lactation which are asserted by some are denied by others; those caused by menstruation, sickness, or ingesta are either well understood or dimly appreciated, but rarely measurable. The nature of its proteid, whether uniform or compound, is not sufficiently known. Whether there is an essential quality that is beyond the domain of chemistry is not known. That is why so many and so different iron clad rules have been established for the selection of substitutes, and why the uncertainty has rendered experimentation with commercial substitutes, by chemists and even by respectable clinicians, so common.

If human milk were a uniform body, the demand for an exactly equivalent substitute would be justified. Nature, however, is more liberal in allowing latitude than a chemist.

Heat both improves and injures milk. When milk is exposed to 68-70° C. ten or fifteen minutes heat destroys the bacterium coli and b. lactis aerogenes; after long exposure, it kills pathogenous germs; at 80° C. it coagulates albumin and changes the taste and odor of the milk. Even at 70° C. it changes casein so as to impair its value for dairy purposes. In boiling, a part of the albumin is deposited, the lecithin is destroyed, the fat altered, both chemically and physically. Serious changes appear to take place, through long boiling, both in casein and in nuclein. High temperatures continued for hours are required to destroy some of the long lived spores; their injuriousness, however, is not entirely clear.

Boiled, pasteurised or sterilised cow's milk is never woman's milk; it is not a curative agent, it affords, however, the great advantage of destroying fermenting and pathogenous germs; that is why it is indispensable in large cities and during the prevalence of certain epidemics, and wherever fresh and unpolluted milk is not accessible. When employed as exclusive infant food, cow's milk, watered or not, is liable to cause constipation, or diarrhea, rickets and scurvy. To preserve its antibacteric effect, heat should be followed by immediate and rapid cooling, but not freezing.

Ample dilution of the artificial food of the infant, and particularly of the newly born, is required because of the heterogeneous composition of cow's milk; even those who are as usual put to an incompetent breast require water to combat loss of weight and the tendency to nephritis and to nephrolithiasis, the former of which is still more frequent than the latter, indeed very frequent. A large amount of liquid does not interfere with the motility of the stomach and does not cause dilatation, firstly because the normal infant is no glutton and secondly because absorption begins at once.

Easy and equal digestibility of the casein in woman's and in cow's milk is either asserted or denied by good observers. When cereal decoctions are mixed with cow's milk its casein is claimed and proven to precipitate in fine tufts. On the other hand, cereals are said to have no such effect any more than water. Water, however, is recommended by the same authors as addition to cow's milk. This contradiction is met with those who recommend dextrinised flours for the dilution of cow's milk. The digestibility of a certain amount of amylum, such as is contained in cereals, has been erroneously and persistently denied when it had long been proven both by experience and by experiments to exist.

Cereal decoctions are the proper diluents for the surplus casein of cow's milk.

Milksugar is partly absorbed in the stomach, partly in the intestine, partly changed into lactic acid. It is required for digestion and is an antiseptic. Not enough of it, however, can be given to have the latter effect to the same degree that is exerted by flours. Besides, milk peptones disappear with acid fermentation. That is why milk-sugar should not be given in large quantities but other carbohydrates, in its stead. Cane sugar should take its place, particularly as for the purposes of digestion there is milk sugar enough in cow's milk that forms part of every artificial food, and because there is a ferment in the intestine of the young which inverts cane sugar and renders it absorbable. Besides, every other carbohydrates has the same power to protect albumin against putrefaction.

Fat is added to cow's milk for the alleged purpose of increasing its nutritive property (by preventing the loss of the fat and albumin of the tissues), and by loosening and separating the minute particles of casein.

It should be remembered, however, that even human milk contains often so much fat as to cause "fat diarrhea," that the normal infant feces eliminate unchanged fat in goodly quantity, that moreover the fat globules of cow's milk are larger, less numerous, and less absorbable than those of woman's milk, that the two fats are not equal chemically, and that the urine is liable after feeding with cow's milk fat to contain ammonium, and the gut toxins.

The mineral constituents of cow's and human milk are different. The addition of chloride of sodium to artificial foods is required both for physiological and chemical reasons.

Home-made artificial foods are preferable to the proprietary foods of the market, for many reasons.

The separation of the component parts of cow's milk by mechanical means and the recomposition of the same is a procedure of doubtful value.

Experience of the physician and of the well directed public at large is equivalent at least to laboratory and library theories based on facts that are sub judice.

### THE PATHOGENY OF GOUT.

By Sir DYCE DUCKWORTH, M. D., L.L. D., London.
Physician and lecturer on medicine St. Bartholomew's Hospital, London.

1. That gout as a morbid condition depends on an inherent vice of nutrition, which is manifested by an imperfect metabolism in various organs or parts of the body, presumably in the kidneys, and probably in the liver.

2. That this trophic disorder or inadequacy (ralentissement de nutrition) leads to the formation of uric acid, probably in excess, and to the periodic retention of it in the blood (gouty uricemia).

3. That histology throws no light upon the intimate nature of this defect which thus relates to cellular potentiality, possibly under neuro-trophic influence, and not, so far as we know, to structural alteration.

4. That this textural disability, or a tendency to it, may be primarily acquired, and also transmitted as a fault, thereby inducing from time to time uricemia with gouty manifestations in the descendants.

5. That in most instances, under conditions which provoke it, and in some cases independently of these, attacks of gout may grow up and come to a crisis. Such crises are attended by an alteration in the solubility of the uratic salt in the blood, whereby irritating crystals of biurate of sodium are produced, and precipitated in various parts of the body.

6. That a paroxysm of gout, the sites of its occurrence, and its metastases, are determined by nervous influences, probably dominated from the bulbar center, and that the local attacks alight either in the joints, or in textures which have been weakened or rendered vulnerable by impaired nutrition, owing to past injury or over-use.

7. That this central neurosis is an essential and transmissible feature in the pathogeny of gout, and pertains to the arthritic diathesis generally.

8. That the uricemia of gout is peculiar and unlike that which is induced by other morbid conditions, but that the occurrence of uricemia in the gouty is by itself inadequate to induce attacks of gout.

9. That uratic deposits in any part of the body may be removed in course of time, but are apt to be permanent in the least vascular tissues.

10. That uratic deposits may occur to an enormous extent in gouty persons without the occurrence of any pain or paroxysms.

11. That the clinical features of gout indicate that both hemic changes (due to inherent morbid tissue metabolism) and a neuro-trophic disturbance act as pathogenic factors, and that, consequently, gout is to be regarded as a neuro-humoral malady.

